# The Ideal Sanitation of a Physician’s Office

**Published:** 1895-12

**Authors:** Bushrod W. James

**Affiliations:** Philadelphia, Pa.


### THE IDEAL SANITATION OF A PHYSICIAN'S OFFICE.

[Read before the Pennsylvania State Homœopathic Medical Society.]

BUSHROD W. JAMES, A. M., M. D., PHILADELPHIA, PA.

When an individual enters the medical profession, he or she accepts the responsibilities which a knowledge of all the branches of medicine, even to that of Hygiene and Sanitary Science, demands, his duty being first, to prevent disease if possible; secondly, to try to cure if he cannot prevent it, and thirdly, to cure with the most efficient remedial agents, and as rapidly and safely as it is possible for him to do in the light of advanced medical science.

Every physician should be well instructed in Hygiene and Sanitary Science, and, having this knowledge, he should apply it: first, personally; next, to his residence; then, to his office. The last would seem to be the least important, but to the physician of our school, where the remedies are usually kept in the office or in a medicine room, in drawers, cases, or closets which are not impervious to dust, smoke, moisture, and odoriferous emanations, the value of excluding as far as possible, or altogether, such objectionable conditions from the corks and mouths of his medicine vials is easily comprehended, for these impurities all contain antidotal or obnoxious qualities and deposits calculated to drop into the medicine contained in the vial when opened.

To a man thoroughly interested in the higher-dilution prescriptions this carelessness would be especially undesirable, and if constantly permitted would certainly upset the value of the remedies prepared on the contact-theory of the higher preparations known as graft preparations, or grafts of pellets mingled with unmedicated pellets in order to gain additional attenuation.

Just here occurs a thought in regard to these higher preparations (some of which are probably twenty, thirty, or fifty years or more, of age), as to whether chemical or other change does not take place in them long before the completion of these periods. Changes of crystallization and deterioration are going on in every department of the organic and inorganic world continually, in the majority of substances, and why should not an alteration result in the very higher preparations, as we find such occurring in the lower attenuations and mother tinctures themselves.

Numerous illustrations of changeable remedies could be given; for instance, Bromine, Iodine, Phosphorus, Spongia, Fluoric Acid, and many of the vegetable tinctures.

Of all of the remedies that are subject to these changes, and at what period after their manufacture for medicinal use these changes occur in some of the remedies, we are as yet quite in the dark, but evidences are frequently apparent that such changes have taken place by the non-action of a remedy when carefully selected on the homœopathic principle, for the simillimum-symptoms in disease.

Sanitarians, therefore, would naturally expect greater care to be exercised from a constant user of these higher preparations, on account of their greater sensitiveness to antidotal room emanations coming in contact with them when the vials are opened.

It is apparent to every one, therefore, that a homœopathic physician, with proclivities for either the higher or lower preparations of medicinal remedies, should exclude from his medicine-room such toxic agencies as tobacco smoke, furnace smoke, gas, or the dust arising from dusting and beating one's clothing, which probably is quite well filled with street dust, (which when examined by the microscope, or analyzed, is found to contain very many obnoxious forms of foreign particles) with which the air is surcharged, and which he foolishly releases by brushing his clothing in the room where his medicines are kept, thereby doing much possible injury to the triturations and tinctures. The emanations from persons who may have their clothing saturated with foul odors of the stable, or the housing of the doctor's wraps and lap-robes in the office, in the winter season, through the night, are likewise objectionable, if they have been left in the stable during a portion of the day, or are wet, or have become muddy or saturated with rain or snow, from the muddy boots or shoes soiled from the street defiling them. No such covering should be allowed to remain to dry out overnight or during the day in the medicine-room of the careful homœopathic prescriber.

There are other points that might be greatly elaborated upon, but I will briefly hint at what he should do and what he should not do in regard to his office. He should see that the perishable medicines are removed occasionally as they become inert, and all surgical instruments and appliances are kept strictly clean. He should have the medicine vial lips and corks dusted with a blower occasionally if dust is found to accumulate upon them, or cleaned with individual pieces of fine tissue-paper to be discarded from each vial.

Scientific men find the prevention of dust from settling upon their specimens a great problem to overcome, especially where the collection is made up of delicate or small articles of exhibit, except by expensive dust-excluding cabinet cases. He should keep out, if possible, all these emanations referred to, and even the odor of plants and flowers like Mentha-p., the Rosaceæ, etc., or such articles as Moschus, Asafœtida, Valerian, Iodoform, Coal-tar or its smoke, asphaltum smoke, or the odors and smoke from a garbage-destroyer, or a soap or bone-boiling factory, etc.

His cuspidor should be cleansed daily, and have some clean water with a disinfectant placed in it and kept in sight, so his consumptive cases will not use the rug or carpet. If his patients expectorate therein, especially if they are tubercular or those with sore throat, chronic nasal or other catarrhs, or have dental abscesses or other purulent discharges, it should be cleaned as soon as they leave.

No surgical waste-dressing should remain in the office after the dressing of a case has been attended to.

The carpet should be swept frequently, and specially in muddy weather, as the street droppings of horses and other deleterious material become ground up in the street dirt or mud, and are carried in on the soles and sides of the shoes in wet weather, and this is particularly the case in the larger towns and cities. Frequent cleansing of articles about the office that can be cleaned is requisite. Good disinfection should frequently be resorted to. In fact, everything should be neat, tidy, and clean. The waste-basket should be emptied daily or oftener if surgical dressings or decomposing articles are thrown therein.

Papers, documents, letters, and circulars should be arranged in order upon the desk, or tables supplied therefor, for the pleasing effect on one's friends upon coming in.

The office should be in as good order and show as much tidiness as if the physician were receiving nothing therein but kings and presidents of republics and millionaire merchantmen.

It is sometimes better to please the family of one multi-millionaire in this way than to satisfy untidy people who, on the principle of "misery loves company," are indifferent to his unsightly or unsanitary office arrangements.

